# Supplementary material for: Schizophrenia-related Xpo7 haploinsufficiency leads to behavioral and nuclear transport pathologies
Source: EMBO Rep. 2025 Jan 7;26(4):948–81. doi: 10.1038/s44319-024-00362-9 (PMC11850608; doi:10.1038/s44319-024-00362-9)
Supplement: Supplementary file 1 — Table EV1 [file 44319_2024_362_MOESM1_ESM.docx]

**Table EV1**

|  | Astro | EX | INH | Micro | Olig | OPC |
| --- | --- | --- | --- | --- | --- | --- |
| 3M_Xpo7^+/+^ | 481 | 3342 | 1772 | 328 | 1562 | 250 |
| 6M_Xpo7^+/+^ | 403 | 2403 | 1239 | 285 | 1150 | 180 |
| 3M_Xpo7^+/-^ | 467 | 3179 | 2120 | 420 | 1166 | 242 |
| 6M_Xpo7^+/-^ | 413 | 2153 | 1638 | 352 | 1469 | 227 |

**Table EV1. The numbers of nuclei analyzed in Figure 4B.**

Astro, astrocytes; EX, excitatory neurons; INH, inhibitory neurons; Micro, microglia; Olig, oligodendrocytes; OPC, oligodendrocyte precursor cell.
