# Supplementary material for: Schizophrenia-related Xpo7 haploinsufficiency leads to behavioral and nuclear transport pathologies
Source: EMBO Rep. 2025 Jan 7;26(4):948–81. doi: 10.1038/s44319-024-00362-9 (PMC11850608; doi:10.1038/s44319-024-00362-9)
Supplement: Supplementary file 9 — Expanded View Figures [file 44319_2024_362_MOESM9_ESM.pdf]

Expanded View Figures

Figure EV1. Genotyping, Xpo7 expression analysis, and brain structure analysis of Xpo7 knockout mice.

(**A**) The result of genotyping PCR of Xpo7 knockout mice. (**B**) The amino acid sequence of Xpo7 (exon 1–6). Six types of peptide fragments (red or blue) were detected from the sequence including 1–199 aa (exons 1–6) from Xpo7$^{+/+}$ and Xpo7$^{+/-}$ mice using LC-MS/MS, but not from Xpo7$^{-/-}$ mice. (**C**) H&E staining of coronal section at the bregma of the brain at 3 months of age. Bar: 1 mm. (**D**) Immunostaining for layer-specific markers, Cux1 and Tbr1, reveals normal cortical layer organization in Xpo7$^{+/-}$ mice at 3 months of age. Bar: 100 µm.

© The Author(s)

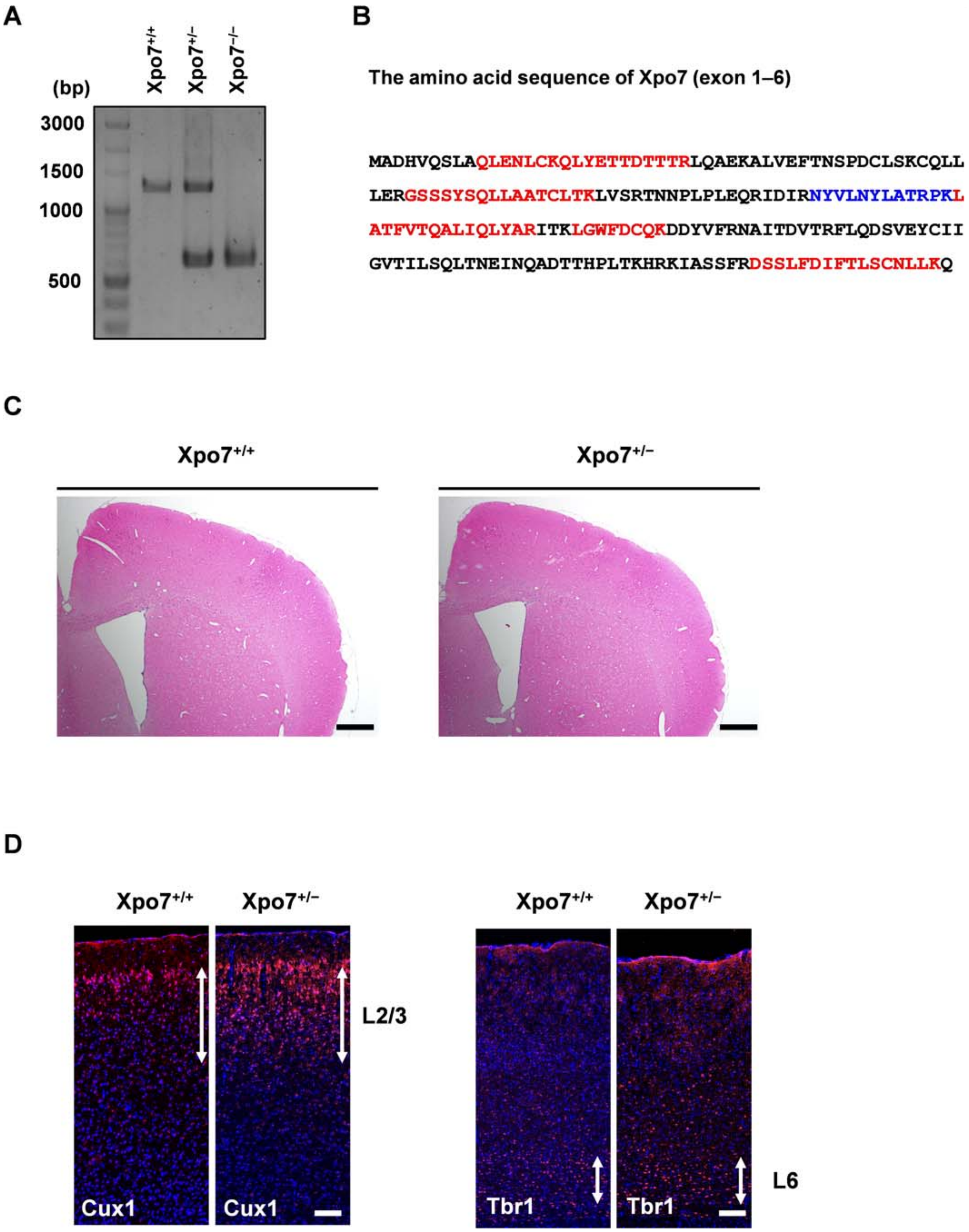
A
(bp)
Xpo7+/+
Xpo7+/−
Xpo7−/−
3000
1500
1000
500
B
The amino acid sequence of Xpo7 (exon 1–6)
MADHVQSLAQLENLCKQLYETTDTTTRLQAEKALVEFTNSPDCLSKCQLL
LERGSSSYSQLLAATCLTKLVSRTNNPLPLEQRIDIRNYVLNYLATRPKL
ATFVTQALIQLYARITKLGWFDCQKDDYVFRNAITDVTRFLQDSVEYCII
GVTILSQLTNEINQADTTHPLTKHRKIASSFRDSSLFDIFTLSCNLLKQ
C
Xpo7+/+
Xpo7+/−
D
Xpo7+/+
Xpo7+/−
L2/3
Cux1
Cux1
Xpo7+/+
Xpo7+/−
L6
Tbr1
Tbr1

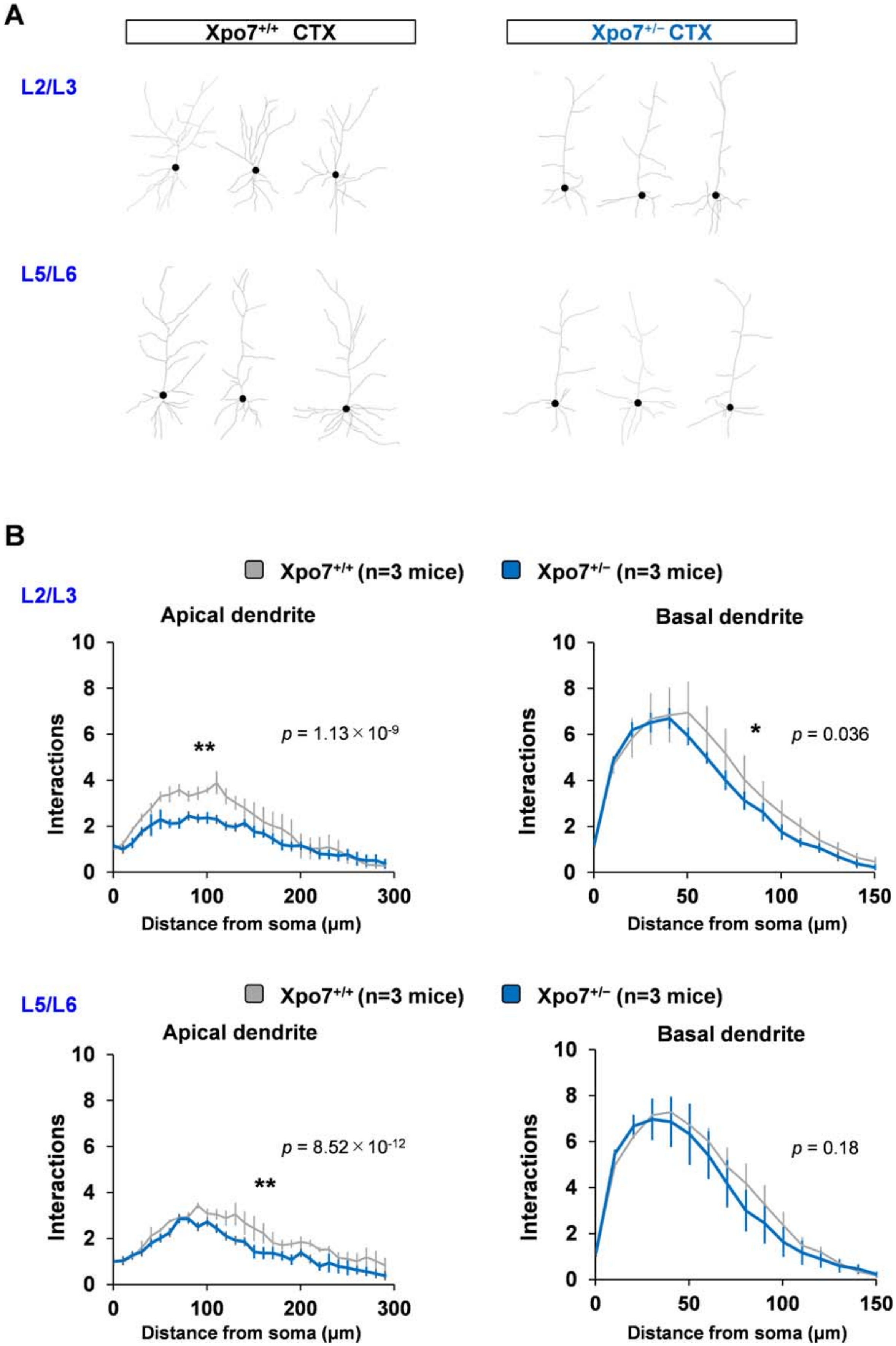
A
Xpo7+/+ CTX
Xpo7+/− CTX
L2/L3
L5/L6
B
Xpo7+/+ (n=3 mice)
Xpo7+/− (n=3 mice)
L2/L3
Apical dendrite
Basal dendrite
Interactions
p = 1.13 × 10-9
**
*
p = 0.036
Distance from soma (μm)
L5/L6
Apical dendrite
Basal dendrite
p = 8.52 × 10-12
**
p = 0.18
Distance from soma (μm)

 © The Author(s)

Figure EV2. Dendrite analyses from the Golgi staining data revealed fewer dendrite branches in Xpo7$^{+/-}$ mice.

(**A**) Representative neurons from L2/L3 and L5/L6 of the frontal cortex at three months of age. (**B**) Sholl analyses of apical and distal dendrites of L2/L3 neurons and L5/L6 neurons of the frontal cortex at three months of age. *$P < 0.05$, **$P < 0.01$ ($N = 3$ mice. For each animal, 6–12 pyramidal neurons from either layers 2–3 or layers 5–6 were analyzed. Two-way repeated ANOVA test). Data are expressed as the mean ± s.e.m.

© The Author(s)

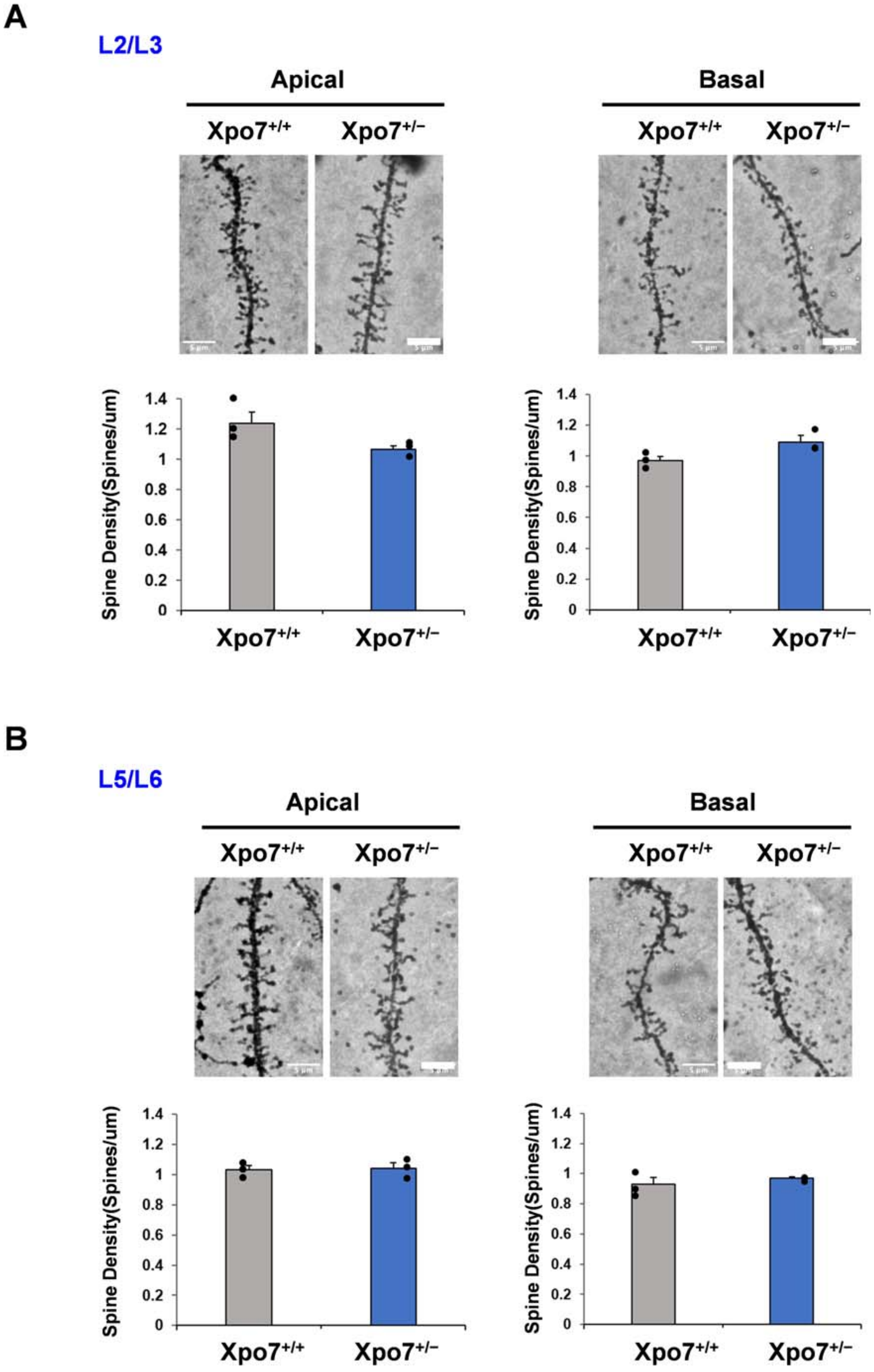
A
L2/L3
Apical
Basal
Xpo7+/+
Xpo7+/−
Spine Density(Spines/um)
1.4
1.2
1
0.8
0.6
0.4
0.2
0
B
L5/L6
Apical
Basal

 © The Author(s)

Figure EV3. Spine analyses from Golgi staining data revealed no significant change in spine densities per dendrite in Xpo7$^{+/-}$ mice.

(**A**) Representative spines from L2/L3 neurons of frontal cortex at three months of age. The graphs show quantitative analysis of spine number. ($N = 3$ mice, 6–14 neurons/mice) Bar: 5 µm. Data are expressed as the mean ± s.e.m. (**B**) Representative spines from L5/L6 neurons of frontal cortex at three months of age. The graphs show quantitative analysis of spine number. ($N = 3$ mice, 6–14 neurons/mice) Bar: 5 µm. Data are expressed as the mean ± s.e.m.

© The Author(s)

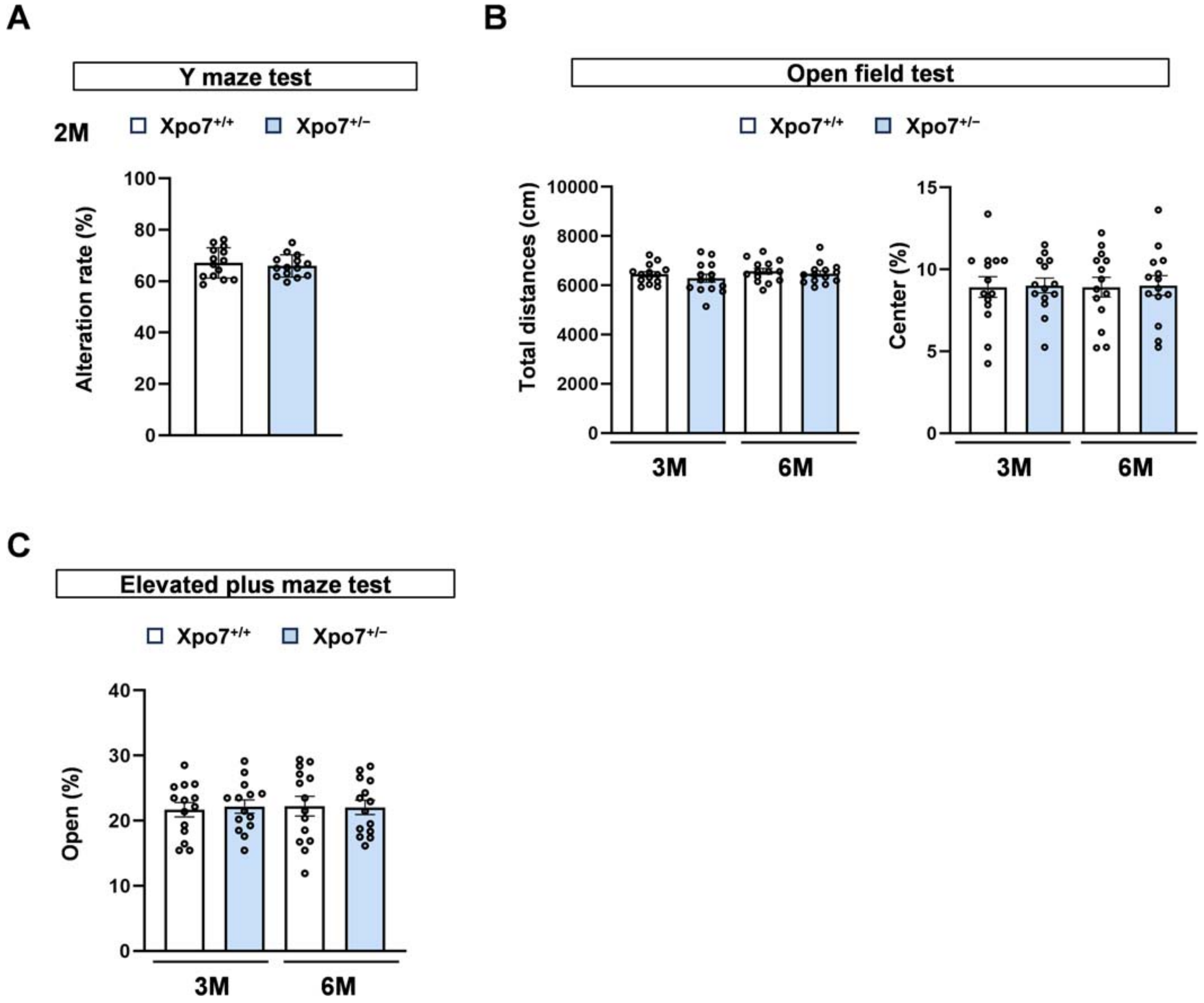

Figure EV4. Behavioral analysis of $XPO7^{+/-}$ mice.

(**A**) Alteration ratios in the Y maze test with $Xpo7^{+/+}$ and $Xpo7^{+/-}$ mice at 2 months of age. There was no significant difference between the groups. ($N = 14$ for each group; Tukey's HSD test). Data are expressed as the mean ± s.e.m. (**B**) Total distances (left) and Time in center region (right) in the open-field test. There was no significant difference between the groups. ($N = 14$ for each group; Tukey's HSD test). Data are expressed as the mean ± s.e.m. (**C**) Time in the open arm regions in the elevated plus maze test. There was no significant difference between the groups. ($N = 14$ for each group; Tukey's HSD test). Data are expressed as the mean ± s.e.m.

 © The Author(s)

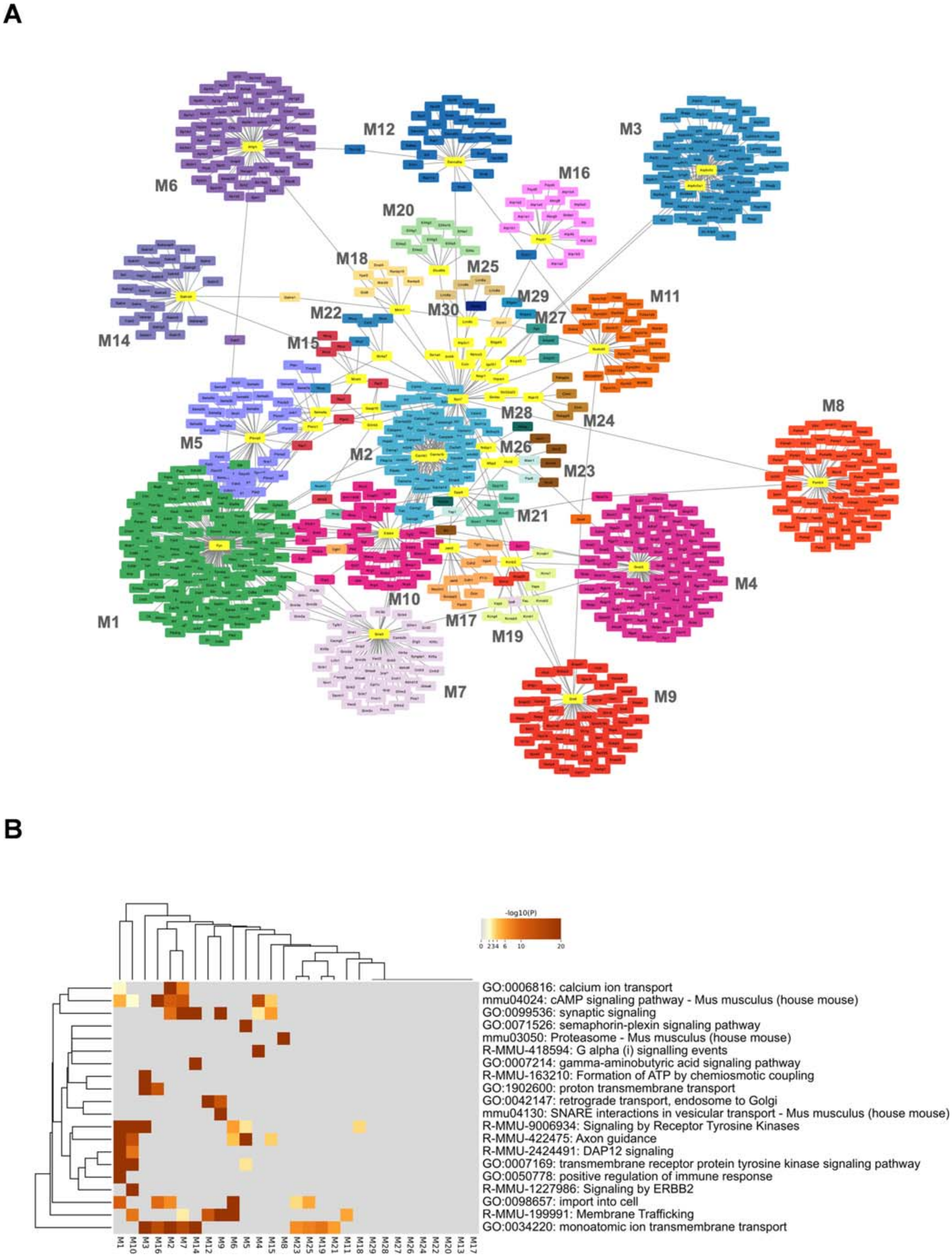
A
M6
M12
M3
M16
M20
M18
M25
M14
M22
M30
M29
M27
M11
M15
M28
M24
M8
M5
M26
M2
M23
M21
M1
M10
M17
M19
M4
M7
M9
B
-log10(P)
0 2 3 4 6 10 20
GO:0006816: calcium ion transport
mmu04024: cAMP signaling pathway - Mus musculus (house mouse)
GO:0099536: synaptic signaling
GO:0071526: semaphorin-plexin signaling pathway
mmu03050: Proteasome - Mus musculus (house mouse)
R-MMU-418594: G alpha (i) signalling events
GO:0007214: gamma-aminobutyric acid signaling pathway
R-MMU-163210: Formation of ATP by chemiosmotic coupling
GO:1902600: proton transmembrane transport
GO:0042147: retrograde transport, endosome to Golgi
mmu04130: SNARE interactions in vesicular transport - Mus musculus (house mouse)
R-MMU-9006934: Signaling by Receptor Tyrosine Kinases
R-MMU-422475: Axon guidance
R-MMU-2424491: DAP12 signaling
GO:0007169: transmembrane receptor protein tyrosine kinase signaling pathway
GO:0050778: positive regulation of immune response
R-MMU-1227986: Signaling by ERBB2
GO:0098657: import into cell
R-MMU-199991: Membrane Trafficking
GO:0034220: monoatomic ion transmembrane transport
M1 M10 M3 M16 M2 M7 M14 M12 M9 M6 M5 M4 M15 M8 M23 M25 M19 M21 M11 M18 M29 M28 M27 M26 M24 M22 M20 M13 M17

© The Author(s)

Figure EV5. Protein interaction network analysis 45 Xpo7 direct-effect molecules.

(**A**) The 1-hop protein–protein interaction analysis with Xpo7 revealed 847 molecules. the Infomap algorithm revealed 28 modules (M1 – M28). (**B**) Gene Ontology (GO) analysis of (**A**). These analyses revealed that 45 XPO7 direct-effect molecules are associated with transmembrane transport (M10, M7, M12, M9, M6), immune response (M1), axon guidance (M1, M10, M6, M5, M15), synaptic signaling (M2, M7, M14, M9, M4, M15) and proteasome (M8).

 © The Author(s)

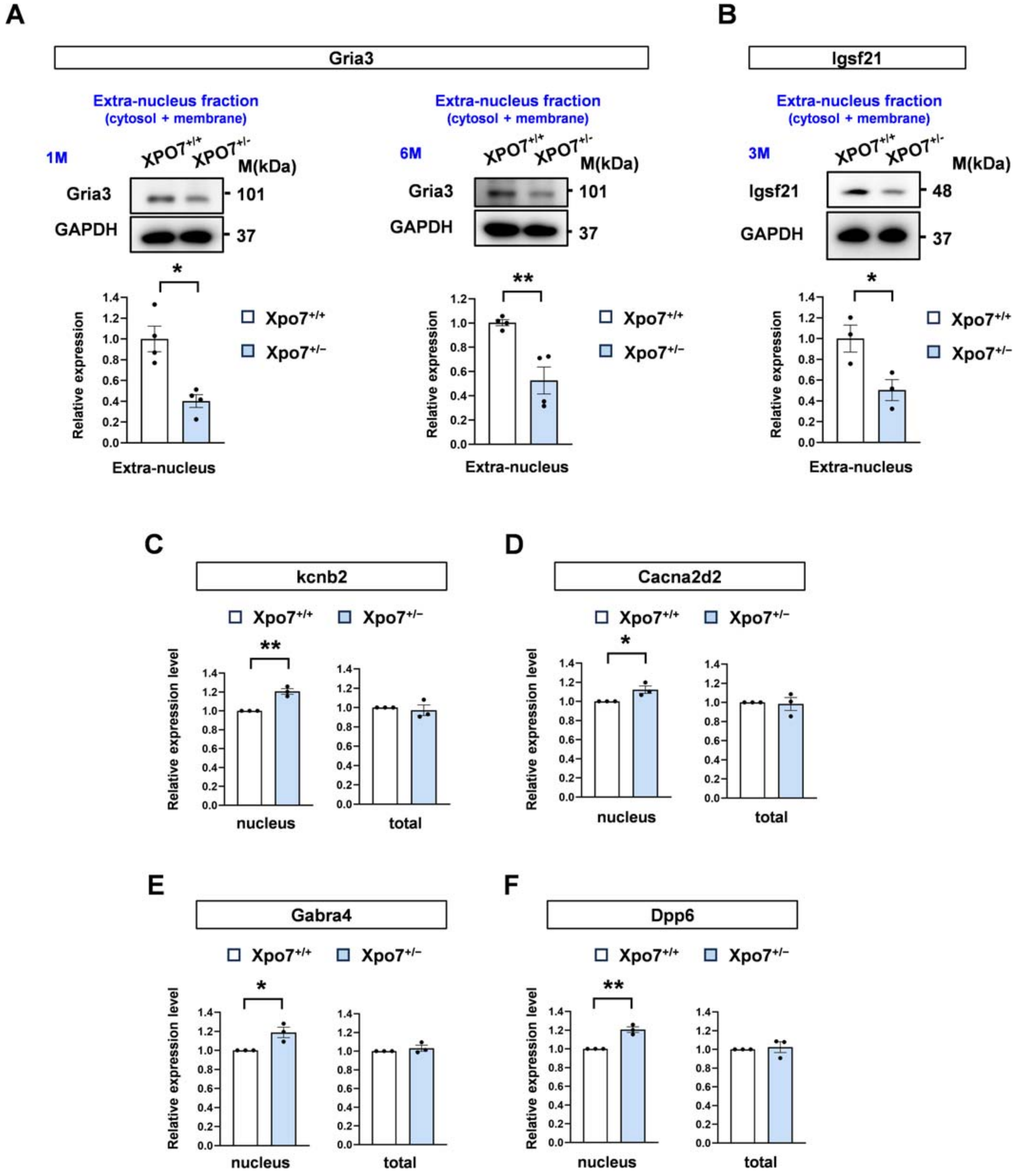
A
Gria3
Extra-nucleus fraction
(cytosol + membrane)
1M
XPO7+/+ XPO7+/-
M(kDa)
Gria3
GAPDH
101
37
Relative expression
Extra-nucleus
6M
B
Igsf21
3M
48
Xpo7+/+
Xpo7+/–
C
kcnb2
Relative expression level
nucleus
total
D
Cacna2d2
E
Gabra4
F
Dpp6

Figure EV6. Detailed analysis of Xpo7-binding and Xpo7 haploinsufficiency-affected molecules.

(**A**) Western blot analysis of Gria3 in the extranuclear fraction from the frontal cortex of $Xpo7^{+/+}$ and $Xpo7^{+/-}$ mice at 1 and 6 months of age. $^{**}P < 0.01$ (1 M $p = 0.0049$; 6 M $P = 0.0057$; $N = 4$ experiments; Tukey's HSD test). Data are expressed as the mean ± s.e.m. (**B**) Western blot analysis of Gria3 in the extranuclear fraction from the frontal cortex of $Xpo7^{+/+}$ and $Xpo7^{+/-}$ mice at 3 months of age. $^{*}P < 0.05$ ($P = 0.039$; $N = 3$ experiments; Tukey's HSD test). Data are expressed as the mean ± s.e.m. (**C–F**) Relative expression of kcnb2, Cacna2d2, Gabra4, and DPP6 in nuclei and whole cells from the frontal cortex of $Xpo7^{+/+}$ and $Xpo7^{+/-}$ mice by LC-MS/MS analysis. $^{*}P < 0.05$, $^{**}P < 0.01$ (kcnb2 $P = 0.0024$; Cacna2d2 $P = 0.035$; Gabra4 $P = 0.024$; Dpp6 $P = 0.0024$; $N = 3$ experiments, 3 mice per group/experiment; Tukey's HSD test). Data are expressed as the mean ± s.e.m. As described in Fig. 3, the LC-MS/MS analysis data were derived from pairs of mice at 1, 3, and 6 months of age.

 © The Author(s)

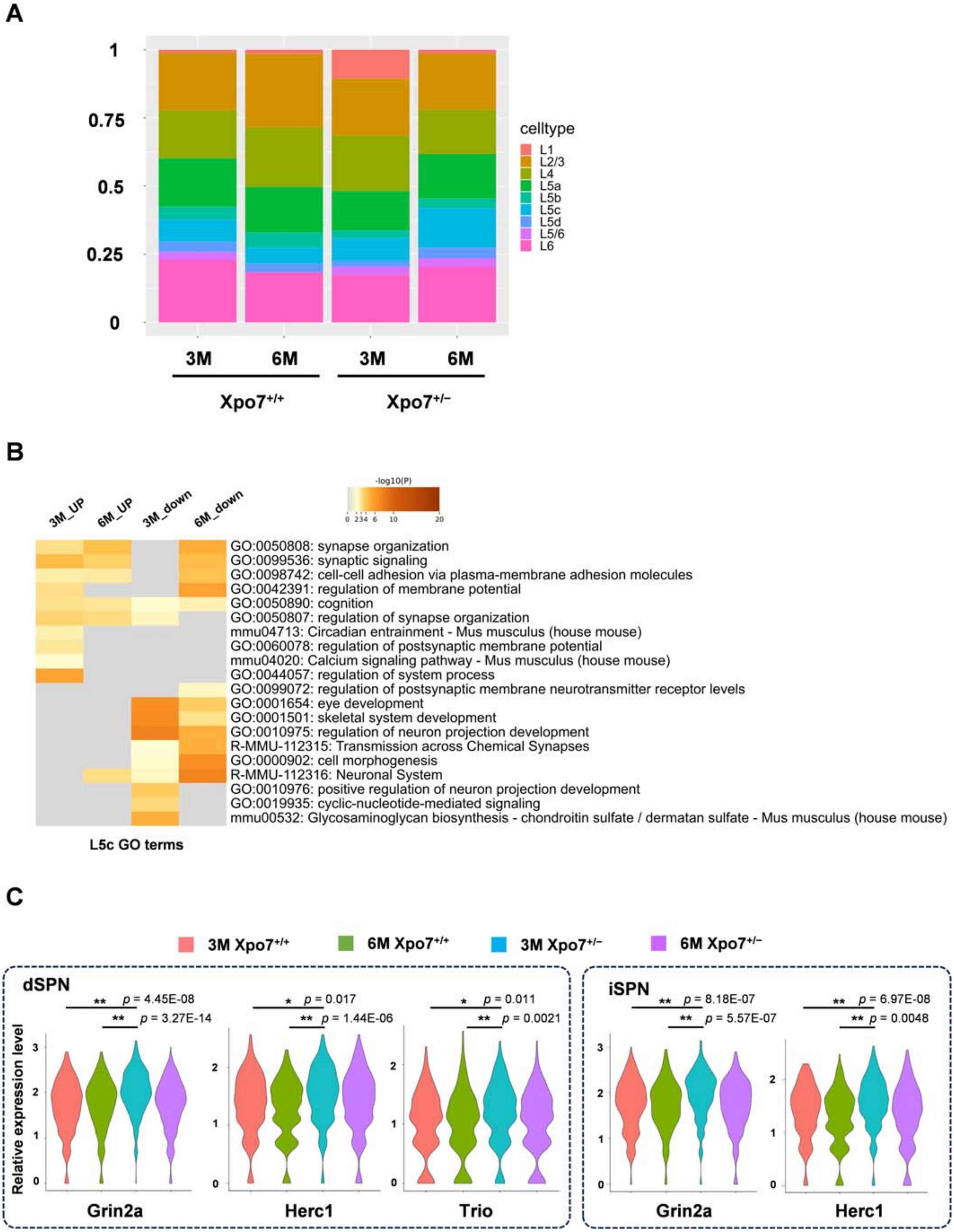
A
1
0.75
0.5
0.25
0
celltype
L1
L2/3
L4
L5a
L5b
L5c
L5d
L5/6
L6
3M
6M
3M
6M
Xpo7+/+
Xpo7+/−
B
3M_UP
6M_UP
3M_down
6M_down
-log10(P)
0 2 3 4 6 10 20
GO:0050808: synapse organization
GO:0099536: synaptic signaling
GO:0098742: cell-cell adhesion via plasma-membrane adhesion molecules
GO:0042391: regulation of membrane potential
GO:0050890: cognition
GO:0050807: regulation of synapse organization
mmu04713: Circadian entrainment - Mus musculus (house mouse)
GO:0060078: regulation of postsynaptic membrane potential
mmu04020: Calcium signaling pathway - Mus musculus (house mouse)
GO:0044057: regulation of system process
GO:0099072: regulation of postsynaptic membrane neurotransmitter receptor levels
GO:0001654: eye development
GO:0001501: skeletal system development
GO:0010975: regulation of neuron projection development
R-MMU-112315: Transmission across Chemical Synapses
GO:0000902: cell morphogenesis
R-MMU-112316: Neuronal System
GO:0010976: positive regulation of neuron projection development
GO:0019935: cyclic-nucleotide-mediated signaling
mmu00532: Glycosaminoglycan biosynthesis - chondroitin sulfate / dermatan sulfate - Mus musculus (house mouse)
L5c GO terms
C
3M Xpo7+/+
6M Xpo7+/+
3M Xpo7+/−
6M Xpo7+/−
dSPN
** p = 4.45E-08
** p = 3.27E-14
* p = 0.017
** p = 1.44E-06
* p = 0.011
** p = 0.0021
Relative expression level
Grin2a
Herc1
Trio
iSPN
** p = 8.18E-07
** p = 5.57E-07
** p = 6.97E-08
** p = 0.0048
Grin2a
Herc1

Figure EV7. Detailed analysis for single-nucleus RNA sequencing analysis.

(**A**) The ratio of neurons in the frontal cortical layers by single-nucleus RNA sequencing analysis. (**B**) GO analysis of DEGs of L5c excitatory neurons. To evaluate the functional enrichment of a given gene list, the metascape performed an accumulative hypergeometric test or Fisher's exact test to calculate *P* values and enrichment factors for each ontology category. (**C**) Relative mRNA expression levels in dSPN and iSPN. Relative mRNA expression levels of Grin2A, Herc1, and TRIO $^{**}P < 0.01$ (dSPN 3 M Xpo7$^{+/+}$ $N = 540$ cells, 6 M Xpo7$^{+/+}$ $N = 544$ cells, 3 M Xpo7$^{+/-}$ $N = 481$ cells, 6 M Xpo7$^{+/-}$ $N = 319$ cells; iSPN 3 M Xpo7$^{+/+}$ N = 442 cells, 6 M Xpo7$^{+/+}$ $N = 487$ cells, 3 M Xpo7$^{+/-}$ $N = 464$ cells, 6 M Xpo7$^{+/-}$ $N = 250$ cells; Wilcoxon rank-sum test).

 © The Author(s)
